# University Pharmacy Clinic: Preventing Errors and Enhancing Lives Through Expert Medication Management

**DOI:** 10.3390/pharmacy13010024

**Published:** 2025-02-10

**Authors:** Alesha Smith, Dhanya Hariharan Nair, Emma R. Smith, Tara F. Wheeler, Lauren E. Smith, Bruce R. Russell, Carlo A. Marra

**Affiliations:** School of Pharmacy, University of Otago, Dunedin 9016, New Zealand; dhanyahari000@gmail.com (D.H.N.); emma.smith@otago.ac.nz (E.R.S.); tara.wheeler@otago.ac.nz (T.F.W.); lauren.e.smith@otago.ac.nz (L.E.S.); bruce.russell@otago.ac.nz (B.R.R.); carlo.marra@curtin.edu.au (C.A.M.)

**Keywords:** medication-related problems (MRPs), pharmacist-led interventions, health-related quality of life (HRQoL), university-based clinics, experiential pharmacy education

## Abstract

The University of Otago School of Pharmacy Clinic serves as a model for innovative medication management, tackling critical medication-related problems (MRPs) to enhance patient outcomes and advance pharmacy education. This study evaluated the clinic’s impact, examining 456 patient consultations over four years, with a focus on MRPs such as dosing errors, non-adherence, and inadequate monitoring. Using the DOCUMENT classification system, pharmacists identified 754 MRPs and issued 836 recommendations, primarily related to medication adjustments and monitoring. Patients reported significant improvements in health-related quality of life, as measured by the SF12V2 survey, with notable gains in mental and physical health metrics. This outcome highlights the clinic’s dual role in optimising patient care and providing pharmacy students with experiential learning opportunities. By integrating hands-on training within a supervised clinical environment, the clinic addresses workforce shortages and reinforces the value of pharmacist-led interventions. The findings advocate for university-based clinics as pivotal hubs for resolving MRPs through interprofessional collaboration, targeted interventions, and innovative technologies such as telepharmacy. The study underscores the need for expanded roles for clinical pharmacists in healthcare policy and practice, showcasing their potential to prevent medication errors, enhance lives, and reshape the future of pharmacy education and patient care.

## 1. Introduction

In the ever-evolving landscape of healthcare, medication management is crucial for ensuring optimal patient outcomes and enhancing the overall quality of care. As the complexity of therapeutic interventions increases, so does the need to identify and resolve medication-related problems (MRPs), which are essential for patient safety. Clinical pharmacists, with their specialised expertise in pharmacotherapy, are pivotal in this process. Their role extends beyond merely dispensing medication—they are key healthcare team members, addressing MRPs and optimising medication regimens to improve patient health outcomes [1,2,3].

To address the shortages of clinical pharmacists, the University of Otago School of Pharmacy Clinic has developed a dedicated hub where expert clinical pharmacists provide patient-centred care focused on identifying and resolving MRPs [4]. Located on the University of Otago campus, the clinic serves a diverse patient population, offering specialised medication management services tailored to various healthcare needs. Whether patients self-refer or are referred by other healthcare professionals, the clinic provides a range of services, including comprehensive medication reviews, medication reconciliation, deprescribing consultations, chronic disease management support, vaccinations, adherence counselling, and ongoing support to maintain effective medication regimens. For example, pharmacists assist patients with complex polypharmacy by optimising their medication regimen, providing smoking cessation programmes, and offering individualised education on managing conditions such as diabetes and hypertension—all at no cost to the patient.

The clinic is not just a teaching facility but a space where learning and practice converge. With dedicated patient interview spaces and the involvement of undergraduate pharmacy students in each consultation, the clinic provides invaluable hands-on experience. These clinical placements allow students to apply their academic knowledge in real-world settings, deepening their understanding of healthcare complexities and honing the competencies required to deliver high-quality patient care. By immersing students in the practical challenges of patient care, the clinic supports the ongoing evolution of the pharmacy profession.

Tackling challenges like funding, patient involvement, and integration into national healthcare systems is essential for sustainable success. Funding constraints may impact staffing and operational capacity, while patient involvement remains vital to ensure consistent participation and adherence to pharmacist recommendations. Additionally, incorporating pharmacist-led clinics into national healthcare frameworks necessitates policy adjustments and collaboration with primary care providers. This study will add to the existing body of evidence by showing how a university-led pharmacist clinic can effectively tackle medication-related issues, improve patient health outcomes, and enhance healthcare efficiency. This research will provide valuable insights for policymakers and healthcare leaders considering the wider implementation of pharmacist-led services by assessing intervention outcomes and pinpointing barriers such as funding limitations and issues with patient involvement.

MRPs remain a significant challenge in healthcare due to their association with increased hospital admissions, adverse drug events, and healthcare costs. Studies indicate that MRPs contribute to nearly 30% of hospital admissions in older adults, highlighting their widespread impact [5]. These problems, which can arise from medication errors, adverse reactions, drug interactions, or non-adherence to prescribed regimens, pose serious risks to patient health. The Pharmaceutical Care Network Europe (PCNE) defines an MRP as “an event or circumstance involving drug therapy that actually or potentially interferes with desired health outcomes”. This definition underscores the importance of identifying and addressing MRPs to ensure safe and effective medication use [1].

The Australian PROMISE II trial systematically classified these medication-related issues using the DOCUMENT classification system. This system was further refined in the PROMISE III trial, enhancing the accuracy and effectiveness of MRP categorization [2,3]. University-led pharmacy clinics in Australia and the United Kingdom have demonstrated positive outcomes in medication management, student training, and reducing medication-related problems (MRPs) [6]. These international examples highlight the potential for pharmacist-led interventions to improve healthcare systems across different regions.

The primary aim of this study was to explore the impact of university clinic pharmacists in preventing medication errors and enhancing patient quality of life through the identification and resolution of MRPs. By analysing data collected during School of Pharmacy clinic consultations, we sought to quantify the prevalence and types of MRPs encountered, evaluate the interventions to prevent these errors, and assess the subsequent improvements in patients’ health-related quality of life.

## 2. Materials and Methods

### 2.1. Study Participants

Ethical approval was obtained from the University of Otago Human Ethics Committee.

All patients visiting the School of Pharmacy Clinic between July 2019 and 30 June 2023 were included in this study; there were no exclusion criteria. Patients were informed that any research utilising their data would only proceed with approval from an ethics committee. They were reassured that any published research would not disclose any personally identifiable information. Despite efforts to recruit patients from diverse populations by advertising and promoting the clinic in various medical centres and local community groups, the pharmacists did not exclude patients based on these criteria. Consequently, the sample may not sufficiently represent New Zealand’s population.

Patients were scheduled for a comprehensive medication review with one of three clinic pharmacists. The consultation length varied based on the patient’s needs, typically lasting up to an hour to thoroughly review medical history, current medications, and any relevant symptoms.

### 2.2. Data Collection

Before the consultation, patients completed a consent form, which included voluntary demographic information, contact details, and consent to a pharmacy student’s presence during the consultation. For phone or Zoom consultations, verbal consent was obtained. The form also gathered details on the patient’s usual pharmacy, general practitioner, any special needs (e.g., hearing, visual impairment, language difficulties), exposure to COVID-19, and present symptoms.

Patients were then offered a validated survey using the Your Health and Well-Being Short Form 12 Version 2 (SF12V2). One month later, a follow-up survey was conducted to monitor health status or adjust medication regimens as needed. Patients completed a satisfaction questionnaire and, upon providing feedback, were entered into a draw for an NZD 50 gift card.

All data were recorded on the REDCap web platform, streamlining survey management. The clinical pharmacists made therapeutic recommendations and advised on lifestyle modifications or other non-pharmacological interventions to enhance health outcomes. These recommendations were communicated to the patient and/or GP through electronic methods (email or patient management system) or paper-based methods (letter) to resolve MRPs. The uptake of the recommendations was not assessed as it fell outside the scope of this study; this is a recognised limitation.

### 2.3. Data Collection Tools

Clinical pharmacists retrospectively reviewed a subset of patient charts (*n* = 103) and classified them according to the DOCUMENT classification system for MRPs. The data were recorded and stored in Excel and REDCap on a secure University server.

Although the study included 456 patients, only 103 patient charts were analysed in detail due to resource constraints and the need for a representative subset for in-depth evaluation. This approach ensured the analysis was thorough while maintaining the study’s validity.

1. The DOCUMENT MRP classification system is a widely used tool for systematically identifying and categorising medication-related problems (MRPs) like adverse reactions, drug interactions, dosing issues, and non-adherence. It provides a structured framework for clinical pharmacists to evaluate and document MRPs, ensures consistency, and allows for precise intervention, optimising patient outcomes. Unlike other tools that may focus solely on adverse drug reactions or compliance, MRPs offer a holistic framework for addressing a broad spectrum of medication issues. Inter-rater reliability was validated through a cross-evaluation process where multiple independent raters classified a subset of MRPs, and Cohen’s kappa statistic was used to measure agreement between raters, confirming their consistency and reliability.
2. The SF12V2 health survey, a shorter version of the SF-36, assesses health-related quality of life by evaluating physical and mental health. It covers eight key health areas and combines scores into two summary measures: the Physical Composite Score (PCS) and the Mental Composite Score (MCS). It was selected for its brevity and reliability in capturing physical and mental health metrics, making it well-suited for this study’s objectives.

### 2.4. Data Analysis

Descriptive statistics characterised patient demographics and baseline treatment. MRPs were assessed using the DOCUMENT classification system, with frequencies reported as percentages. Medicine frequency was analysed using Anatomical Therapeutic Chemical (ATC) codes.

SF12V2 survey scores for physical and mental health were analysed for mean and standard deviation, with a one-month follow-up assessing changes over time. The minimum clinically important difference (MCID) was set at 0.5 times the standard deviation, and Cohen’s effect size (d) quantified observed changes. Statistical analyses were performed using R version 4.1.2/R Studio (2023.03.0).

## 3. Results

### 3.1. Study Population

The study included 456 individuals: 225 males, 222 females, and 9 unspecified. The average age was 68 years (SD 14.18). Ethnically, most were New Zealand European/Pakeha (89%). Most referrals were self-referrals (35%), followed by other healthcare providers (19%). Detailed demographics are shown in Table 1.

### 3.2. Medicine-Related Problems (MRPs)

A review of 103 medication charts revealed 754 MRPs (Table 2). Common issues included non-laboratory monitoring (16%) and patient requests (16%). Other problems included medicine supply and quality issues (8%), dosing errors (too high: 3%; too low: 4%), adherence issues (underuse: 4%; erratic use: 1%), undertreated (9%) and untreated conditions (4%), and potential adverse reactions (1%). Laboratory monitoring was needed for 10% of patients.

### 3.3. Recommendations

Pharmacists issued 836 recommendations (Table 3). The most frequent were changes in medications (23%), non-laboratory monitoring (15%), and laboratory monitoring (12%). Other recommendations included dose adjustments (increase: 5%; decrease: 3%), changes in therapy (2%), referrals (14%), and educational or information provision (24%). In two cases, no recommendation was deemed necessary.

### 3.4. Medicines Implicated in MRPs

In the review of 103 patient charts, 925 medications were assessed, averaging about 9 per chart. The most frequently implicated drugs were cholecalciferol, paracetamol, atorvastatin, omeprazole (each 5%), and aspirin (3%). Other drugs included ibuprofen, bisoprolol, salbutamol, cilazapril, levothyroxine, metoprolol (2% each), and various others, comprising 1% of the total, as shown in Table 4.

### 3.5. SF-12V2 Survey Results

Patients showed improved health-related quality of life one month after clinical pharmacist reviews. General health scores increased, indicating better self-reported health. Physical functioning slightly declined, from 44.77 to 43.98, suggesting a minor decline in performing physical tasks. The Role Physical (RP) score, which measures how much work and other activities were accomplished, increased, indicating an improvement in the participants’ ability to complete tasks. The Body Pain (BP) scores also improved, suggesting a reduction in the participants’ pain level. The mean energy level increased, and participants’ ability to cope with physical or emotional issues also improved, as indicated by the increase in average calmness and depression (MH) score. Overall, these changes reflect a positive impact on patients’ health and well-being.

### 3.6. Physical Composite Score and Mental Composite Score

The results in Table 5 indicate that the mean physical composite score in SF-12V2 at baseline was 32.42 (SD19.44), which increased to 36.08 (SD19.29) after one month. SF-12V2 scores range from 0 to 100, with higher scores indicating better health status. Similarly, the mean mental composite score at baseline was 44.08 (SD18.59), which increased to 48.86 (SD17.74) after the one-month follow-up period. These findings suggest a notable improvement in both physical and mental health over the one-month period (Table 6).

The summary scores in Table 6 demonstrate a significant change in both the Physical (PCS) and Mental Health scores. Specifically, 39% of respondents exhibited an improvement in their PCS, with 22% achieving a change that exceeded the MCID of 9.68. Moreover, 45% experienced a positive change in MCS, with 33% achieving a change that surpassed the MCID value of 9.08. The effect size for the PCS was 0.18, indicating a small effect, while for the MCS, it was 0.26, indicating a small-to-moderate effect. The observed decline in physical discussion could be attributed to the advanced age and multimorbidity of the study population, natural disease progression, or other underlying health conditions not directly addressed by pharmacist interventions

## 4. Discussion

This study aimed to explore the impact of university clinic pharmacists in preventing medication errors and enhancing patient quality of life. The study demonstrates the important role of clinical pharmacists in addressing medication-related problems (MRPs) that persist despite regular interactions with general practitioners (GPs) and community pharmacists. By identifying significant MRPs and providing targeted recommendations to patients’ GPs, clinic pharmacists enhanced patients’ quality of life, making pharmacy clinics accepting self-referrals and practitioner-based referrals an ideal setting for resolving these issues.

### 4.1. Key Clinical Findings

The study found that the most common of the 745 identified MRPs were related to medicine selection, monitoring issues, and patient requests for information. This echoes findings from similar studies in Australia and the Netherlands, highlighting the ongoing need for focused medication management to address these frequent problems [8,9].

The study also revealed that 8% of medication-related problems (MRPs) were linked to medication non-adherence, including underuse, overuse, and improper use. This finding aligns with international research, which has shown that non-adherence can lead to adverse outcomes, and highlights the need for strategies to simplify medication regimens [10,11]. Additionally, 23% of MRPs identified in this study involved patient education, emphasising the importance of empowering patients with knowledge about their medications to prevent non-adherence and improve outcomes.

Clinical pharmacists are ideally placed to help with these errors, as seen in the 836 recommendations, which are primarily related to monitoring and medication adjustment. Previous studies have demonstrated the effectiveness of such pharmacist-led interventions in improving compliance and mitigating drug-related risks [12,13].

The SF12V2 survey results showed a significant improvement in physical and mental health one month after the intervention, suggesting that even brief pharmacist-led interventions can have a meaningful impact on patient well-being.

### 4.2. Implementing These Findings into Routine Practice

The study’s findings advocate for continued collaboration between pharmacists and other healthcare professionals to optimise patient care and outcomes. Given the current workforce and funding shortages for clinical pharmacists in New Zealand and internationally, university-based clinics could effectively serve as hubs for managing MRPs and implementing interventions. This can be achieved through collaboration, technology-driven solutions, targeted interventions, resource optimisation, and advocacy [14,15].

Collaboration is crucial in distributing the workload effectively, particularly in university-based clinics. These clinics offer a unique environment where pharmacy students, interns, and residents can be integrated into the clinical workflow under the supervision of experienced pharmacists. By involving these learners, the burden on clinical pharmacists is reduced, allowing them to focus on more complex tasks while students gain hands-on experience in medication management and patient care. This approach not only enhances the students’ educational experience but also contributes to the overall efficiency and effectiveness of the clinic. Research shows that involving pharmacy students in clinical settings can significantly aid in managing medicine-related problems [6,16]. By providing these opportunities, university clinics can become hubs for both patient care and professional development, ensuring that resources are used efficiently even in the face of workforce and funding challenges.

This research highlights that University clinics should focus their efforts on high-risk patients, such as those with multiple chronic conditions or recent hospital discharges, ensuring that limited resources are used effectively. Standardised protocols for MRP management can streamline processes and allow even less experienced staff or students to contribute meaningfully [17]. Technology, such as telepharmacy and Clinical Decision Support Systems (CDSS), can extend the reach of clinical pharmacists and improve efficiency. Despite being geographically separated, telepharmacy would enable remote consultations and follow-ups, while CDSS helps prioritise critical cases by identifying potential MRPs like drug interactions or high-risk patients [18,19].

### 4.3. Study Limitations

One of the primary limitations of this study is the potential bias introduced by the self-referred patient population. Patients who actively seek pharmacist-led medication reviews may be more engaged in their healthcare, leading to an overrepresentation of individuals with higher adherence to prescribed therapies. This could impact the generalisability of the findings to broader patient populations who may not actively seek such services.

Another key limitation is that the study was conducted within a single university-affiliated pharmacy clinic. While this setting provides a controlled environment for assessing pharmacist interventions, it limits the ability to extrapolate the results to other clinical settings, such as community pharmacies or hospital-based pharmacy services. Future studies should consider multicentre trials to evaluate the effectiveness of pharmacist-led interventions across diverse practice settings.

Lastly, the study did not account for long-term patient outcomes beyond the follow-up period, making it difficult to assess the sustained impact of pharmacist interventions on medication-related problems and health outcomes. Further research should focus on longitudinal studies to track patient progress over extended periods.

### 4.4. Policy Implications

The results of this study carry significant implications for healthcare professionals and policymakers alike. The evidence suggests that university-based clinical pharmacists’ interventions, particularly in identifying and addressing medication-related problems (MRPs), can significantly improve both physical and mental health outcomes.

Incorporating these pharmacist-led strategies into routine healthcare practice is not only beneficial but essential. However, sustainable funding models need to be established, either through government reimbursement schemes or partnerships with private healthcare providers. This would enable pharmacist-led clinics to expand their services without financial barriers. Advocacy for the role of clinical pharmacists in university clinics is crucial in securing support and funding while also necessitating further studies that demonstrate the substantial impact pharmacists have on patient outcomes and, likely, healthcare costs.

Additionally, there is a need to develop standardised metrics for assessing the impact of clinical pharmacists on MRPs and patient outcomes to ensure their contributions are consistently recognised and valued. Regulatory frameworks should adapt to acknowledge pharmacists as essential healthcare providers capable of delivering primary care services. This may include expanding pharmacist prescribing rights and integrating their services into national healthcare strategies.

Finally, policies should encourage interdisciplinary collaboration between pharmacists, general practitioners, and allied healthcare professionals. Establishing clear referral pathways and shared decision-making models would strengthen the integration of pharmacist-led services into mainstream healthcare systems, ultimately improving patient outcomes and reducing healthcare costs.

## 5. Conclusions

In summary, clinical pharmacists in university clinics play a critical role in managing medication-related problems, significantly improving patient outcomes. Their specialised knowledge, collaborative approach, and patient-centred focus make them indispensable healthcare team members. As the healthcare landscape continues to evolve, the role of clinical pharmacists should be further integrated and expanded to maximise their positive impact on patient care.

Several actionable steps should be considered to scale the university clinic model to other settings or healthcare systems. Firstly, creating standardised protocols for pharmacist-led interventions can facilitate their implementation across various clinical environments. These protocols should be adaptable to different healthcare settings, ensuring consistency in service delivery while allowing for flexibility based on local needs.

Additionally, fostering partnerships between universities, healthcare institutions, and policymakers can facilitate knowledge exchange, training opportunities, and research collaborations. Establishing networks for sharing best practises and evaluating outcomes across multiple sites will contribute to the ongoing evolution of pharmacist-led clinics.

Lastly, targeted workforce development initiatives, including training programmes for pharmacy students and continuing education for practising pharmacists, will help them to obtain the necessary skills and competencies for expanding these models beyond academic settings.

## Figures and Tables

**Table 1:** Demographic details from the Pharmacy Clinic data.

Characteristics	Count (*n* = 456)	%
Gender		
Male	225	49
Female	222	49
Missing	9	2
Age, Mean (SD)	67.89 (14.81)	
<25	6	1
25–34	13	3
35–54	47	10
55–64	73	16
65+	289	63
Missing	28	6
Ethnicity ^1^		
New Zealand European Other/Pakeha	407	89
Māori	12	3
Pacific	8	2
Asian	5	1
Referrals *		
Self-referral	159	35
From general practitioners	36	8
From hospital	74	16
From pharmacist	9	2
From other healthcare providers	87	19
From nurse	11	2

^1^ Respondents can select more than one ethnicity, so results may add up to more than 100%. * 80 unknown sources for referrals to the clinic and/or follow-up cases.

**Table 2:** Medicine-related problems (MRPs) (*N* = 754) identified from the 103 reviewed patients.

Code	Category	Sub-Category	Number (% of Category)	Number (% of Total)
D	Drug selection	Duplication	15 (1.98)	129 (17.10%)
		Drug interaction	11 (1.45)	
		Wrong drug	5 (0.66)	
		Incorrect strength	1 (0.13)	
		Inappropriate dosage form	1 (0.13)	
		Contraindications apparent	1 (0.13)	
		No indication apparent	18 (2.38)	
		Other drug selection problem	77 (8.8)	
O	Over- or underdose	Prescribed dose too high	19 (2.51)	63 (8.35%)
		Prescribed dose too low	27 (3.71)	
		Incorrect/unclear dosing instructions	1 (0.13)	
		Other dose problem	16 (2.12)	
C	Compliance	Under use by patient	28 (3.71)	58 (7.69%)
		Overuse by patient	1 (0.13)	
		Erratic use of medication	9 (1.19)	
		Intentional drug misuse	1 (0.13)	
		Difficulty using dosage form	0 0	
		Other compliance problem	19 (2.51)	
U	Undertreated	Condition undertreated	69 (9.15)	114 (15.11%)
		Condition untreated	33 (4.37)	
		Preventative therapy required	4 (0.53)	
		Other undertreated problem	8 (1.06)	
M	Monitoring	Laboratory monitoring	76 (10.07)	203 (26.92%)
		Non-laboratory monitoring	123 (16.31)	
		Other monitoring problem	4 (0.53)	
E	Education or Information	Patient requests drug information	121 (16.04)	176 (23.34%)
		Patient requests disease management advice	45 (5.96)	
		Confusion about therapy or condition	0 (0)	
		Demonstration of device	0 (0)	
		Other education or information problem	10 (1.32)	
N	Not classifiable	Clinical interventions that cannot be classified under another category	2 (0.26)	2 (0.26%)
T	Toxicity or ADR	Toxicity/ADR	9 (1.19)	9 (1.19%)

Adapted from Williams et al. (2012) [3], the original table included the description of Category, Sub-category, described by the DOCUMENT system with the number and percentage of categories and the moderate to high clinical significance of the PROMISE III results, while this table included the number and percentage of categories of pharmacy clinic data. Co-morbidities, medication use, and patient characteristics, such as age, may also influence the occurrence of DRPs.

**Table 3:** Recommendations described by the DOCUMENT system (*N* = 836).

Recommendation		Sub-Category (%)	Category (%)
A change in therapy	Dose increase	44 (5.26)	296 (35.40)
	Dose decrease	23 (2.75)	
	Drug change	191 (22.84)	
	Drug formulation change	3 (0.35)	
	Drug brand change	0 (0)	
	Dose frequency/schedule change	18 (2.15)	
	Prescription not dispensed	0 (0)	
	Other changes to therapy	17 (2.03)	
A referral required	Refer to prescriber	101 (12.08)	118 (14.11)
	Refer to hospital	5 (0.59)	
	Refer to medication review	0 (0)	
	Other referral required	12 (1.43)	
Provision of information	Education/counselling session	100 (11.96)	198 (23.68)
	Written summary of medications	21 (2.51)	
	Commence dose administration aid	0 (0)	
	Other written information	77 (9.21)	
Monitoring	Monitoring laboratory test	97 (11.60)	222 (26.55)
	Monitoring: non-laboratory	125 (14.95)	
Other	No recommendation necessary	2 (0.24) *	
Total		836 (100.0%)	

* Not included in the recommendation analysis. Adapted from Williams et al. (2012), the original table included the recommendations described by the DOCUMENT system [3].

**Table 4:** Top 10 ATC medicines groups classified.

ATC CODE	Description	Frequency of Drugs (*N* = 925)	Total, %
A11CC05	Vitamin D and analogues	Cholecalciferol (48)	5.18
N02BE01	Anilides, analgesics, other analgesics, and antipyretics	Paracetamol (48)	5.18
C10AA05	Lipid-modifying agents/HMG co a reductase inhibitors	Atorvastatin (46)	5.00
A02BC01	Proton pump inhibitors	Omeprazole (43)	4.64
N02BA01	Analgesic and antipyretics	Aspirin (26)	2.81
M01AE01	Anti-inflammatory and antirheumatic products, non-steroids	Ibuprofen (20)	2.16
C07AB07	Beta-blocking agents, selective	Bisoprolol (18)	1.94
R03AC02	Drugs for obstructive airway disease/adrenergic inhalants	Salbutamol (15)	1.62
C09AA08	Agents acting on the renin–angiotensin system/ace-inhibitors, plain	Cilazapril (14)	1.51
H03AA01	Thyroid therapy	Levothyrozine (14)	1.51
C07AB02	Beta-blocking agents, selective	Metoprolol (14)	1.51
C09CA06	Cardiovascular system/agents acting on the renin–angiotensin system	Candesartan (13)	1.40
N02BF01	Analgesics and antipyretics	Gabapentin (13)	1.40
MO4AAO1	Antigout preparations/preparations inhibiting uric acid production	Allopurinol (11)	1.18
C03CA01	Diuretics/high-ceiling diuretics/loop diuretics	Furosemide (11)	1.18
C02CA04	Antihypertensives/antiadrenergic agents, alpha-adrenoreceptor antagonists	Doxazosin (11)	1.18
N06AX16	Antidepressants	Venlafaxine (11)	1.18
Total drugs prescribed		925	100

**Table 5:** SF-12V2 health survey results (*N* = 97).

Domain	Mean Score (SD)	Mean Score (SD) After 1 Month Follow-Up	*p* Value	Effect Size Cohen’s d
General Health (GH)	44.66 (24.08)	48.85 (22.91)	0.027	0.18
Physical Functioning (PF)	44.77 (36.28)	43.98 (38.34)	0.6937	0.03
Role Physical (RP)	49.95 (29.29)	54.40 (28.90)	0.17	0.14
Body Pain (BP)	55.34 (32.57)	57.71 (30.98)	0.3426	0.07
Vitality (VT)	40.23 (27.13)	45.75 (24.51)	0.0007538	0.30
Social Functioning (SF)	67.08 (37.55)	72.22 (28.04)	0.412	0.08
Role Emotional (RE)	67.02 (27.83)	74.18 (25.54)	0.007467	0.25
Mental Health (MH)	64.78 (21.66)	67.52 (19.93)	0.1566	0.13

GH = health; PF = moderate_activities + climbing_stairs; RP = accomplish_less + work_other_activites; BP = pain; VT = energy; SF = physical_emotional; RE = accomplish_less_emotion + activities_less_carefully; MH = calm + depressed.

**Table 6:** Physical and mental scores (*N* = 97).

Scores	Pre-Visit (SD)	Post-Visit (SD)	Change	MCID (0.5 × SD ^+^)	Effect Size (d)
P score	32.42 (19.44)	36.08 (19.29)	−3.66	9.68	0.18
M score	44.08 (18.59)	48.86 (17.74)	−4.78	9.08	0.26

MCID formula is taken from Norman et al. [7]. + denote pooled standard deviation. Effect size (d) Cohen’s d was calculated using (post pscore − pre pscore)/pooled SD.

## Data Availability

Due to clinical privacy, data are not available.

## Abbreviations

The following abbreviations are used in this manuscript:

MRPs	Medication-related problems
PCNE	Pharmaceutical Care Network Europe
PROMISE	Program of Research on Medication Issues and Solutions Evaluation
DOCUMENT	Drug-related problems Outcomes and Categorisation and Utility Evaluation in Clinical Tools
SF12V2	Short-Form 12 Version 2 (Health Survey)
PCS	Physical Composite Score
MCS	Mental Composite Score
ATC	Anatomical Therapeutic Chemical (classification system)
CDSS	Clinical Decision Support Systems
GP	General Practitioner
MCID	Minimum Clinically Important Difference
ADR	Adverse Drug Reaction
